# Pulmonary Toxicity after Total Body Irradiation—An Underrated Complication? Estimation of Risk via Normal Tissue Complication Probability Calculations and Correlation with Clinical Data

**DOI:** 10.3390/cancers13122946

**Published:** 2021-06-12

**Authors:** Michael Oertel, Christopher Kittel, Jonas Martel, Jan-Henrik Mikesch, Marco Glashoerster, Matthias Stelljes, Hans Theodor Eich

**Affiliations:** 1 Department of Radiation Oncology, University Hospital Muenster, 48149 Muenster, Germany; christopher.kittel@ukmuenster.de (C.K.); j_mart18@uni-muenster.de (J.M.); marco.glashoerster@ukmuenster.de (M.G.); hans.eich@ukmuenster.de (H.T.E.); 2 Department of Medicine A—Hematology, Hemostaseology, Oncology, Pulmonology, University Hospital Muenster, 48149 Muenster, Germany; jan-henrik.mikesch@ukmuenster.de (J.-H.M.); matthias.stelljes@ukmuenster.de (M.S.)

**Keywords:** total body irradiation, stem cell transplantation, lung toxicity, survivorship

## Abstract

**Simple Summary:**

Total body irradiation is an integral part of many conditioning regimens prior to allogeneic stem cell transplantation. It is a large-field technique affecting all organs at risk, of which the lungs are critical for patient survival. However, the precise rates of long-term pulmonary toxicities are unknown. This analysis provides a large patient cohort with long-term follow-up investigating TBI sequelae. Additionally, we present normal tissue complication probability calculations for acute and chronic lung toxicities to enable comparison between biophysical and real-world data. To our knowledge, this is the first adaption of this model to a total-body irradiation patient cohort, which will help to evaluate the feasibility and appropriateness of this approach.

**Abstract:**

Total body irradiation (TBI) is an essential part of various conditioning regimens prior to allogeneic stem cell transplantation, but is accompanied by relevant (long-term) toxicities. In the lungs, a complex mechanism induces initial inflammation (pneumonitis) followed by chronic fibrosis. The hereby presented analysis investigates the occurrence of pulmonary toxicity in a large patient collective and correlates it with data derived from normal tissue complication probability (NTCP) calculations. The clinical data of 335 hemato-oncological patients undergoing TBI were analyzed with a follow-up of 85 months. Overall, 24.8% of all patients displayed lung toxicities, predominantly pneumonia and pulmonary obstructions (13.4% and 6.0%, respectively). NTCP calculations estimated median risks to be 20.3%, 0.6% and 20.4% for overall pneumonitis (both radiological and clinical), symptomatic pneumonitis and lung fibrosis, respectively. These numbers are consistent with real-world data from the literature and further specify radiological and clinical apparent toxicity rates. Overall, the estimated risk for clinical apparent pneumonitis is very low, corresponding to the probability of non-infectious acute respiratory distress syndrome, although the underlying pathophysiology is not identical. Radiological pneumonitis and lung fibrosis are expected to be more common but require a more precise documentation by the transplantation team, radiologists and radiation oncologists.

## 1. Introduction

Total body irradiation (TBI) is an effective conditioning modality before allogeneic stem cell transplantation (alloSCT) in the treatment of acute leukemias [1,2]. With its application not being influenced by either pharmacodynamic or -kinetics or blood supply, it may complement chemotherapy as a conditioning agent and address putative sanctuary sites such as the brain or testes [1,2]. This efficacy has to be carefully balanced with (long-term) side effects, of which pulmonary toxicity may impair both quality of life and survival.

Radiation-induced lung toxicities are caused by a complex mechanism involving damage to the alveolar epithelia, cell senescence, oxidative stress and local inflammation (pneumonitis) [3,4,5]. With the attraction of fibroblasts and collagen deposition, subacute pneumonitis is superseded by chronic lung fibrosis. The clinical presentation is variable, comprising asymptomatic courses but also acute and/or chronic respiratory insufficiency leading to intensive care and/or need for supplemental oxygen [3,5,6].

Data on the incidence of pneumonitis after TBI vary depending on the patient cohort and treatment technique, covering a range of 10.3–45% [7,8,9,10,11,12,13,14,15]. However, clinical cohorts are often small and bear limited follow-up, thus not allowing multivariate analysis and probably underestimating the long-term side effects. This hampers a precise estimation and understanding of lung toxicities, which is crucial for mediastinal irradiation.

The present analysis aims at providing a detailed evaluation of long-term pulmonary toxicities in a large patient cohort treated at a single institution. It is complemented by a biophysical evaluation of a normal tissue complication probability (NTCP) model that calculates radiotherapy (RT) toxicity likelihood and allows for comparisons between estimated data and observed side effects. This theoretical approach offers the possibility to calculate and thereby anticipate the rate of pulmonary toxicities for a given RT regimen in order to establish a risk analysis. NTCP calculations have been used successfully for other entities [16,17] but have not been applied to a TBI cohort yet. In addition, a comprehensive discussion on influence factors for pulmonary toxicities is provided.

## 2. Materials and Methods

### 2.1. Clinical Data

We analyzed patients who underwent conditioning regimens containing TBI that preceded alloSCT at our institution between 2001 and 2018. After TBI and SCT, follow-up was carried out according to the guidelines of the European Leukemia Net and the “Deutsche Arbeitsgemeinschaft für Hämatopoetische Stammzelltransplantation und Zelluläre Therapie e.V.”. Patient data were received from clinical files. In case of unavailability of data, family doctors were contacted to receive additional information. A minimum follow-up of 1 year was required to account for long-term toxicity. Toxicities were graded using the national cancer institute’s common terminology criteria for adverse events version 5.0 [18].

### 2.2. Statistical Analysis

For statistical analysis, the program SPSS^®^ version 27.0 (IBM^®^, Armonk, NY, USA) was used. Time-dependent event and incidence curves were generated using the Kaplan–Meier method. The interval between treatment and the onset of a pulmonary toxicity was described as pulmonary toxicity-free survival (PTFS), which was determined as the time between the first day of RT to the respective event. Comparisons between categorical variables were made with the log-rank test, with a *p*-value below 0.05 considered to be statistically significant. The Cox proportional hazards model was applied to determine the relative risk of pulmonary toxicities both as a univariate and a multivariate analysis (backward elimination (likelihood ratio)), the latter being used for variables with a *p*-value below 0.15 in the univariate analysis. To analyze the association between different risk factors and the grade of pulmonary toxicities, the exact Fisher–Freeman–Halton test was used for categorical variables and the Mann–Whitney-U test for the risk factor “age”.

### 2.3. Planning

Radiation planning was adapted to the anterior–posterior diameter measured subcostally in the median sagittal plane. Beginning in 2018, all patients received a planning CT, enabling individual planning. Three-dimensional conformal radiotherapy plans were created on the Eclipse planning system version 15.6 (Varian Medical Systems, Palo Alto, CA, USA). In order to accommodate the positioning of the patient (four lying positions), different beam angles were used in the planning process (anterior to posterior, posterior to anterior and two lateral beams). Planning was executed via the AAA algorithm. A support structure representing the beam spoiler was added into the path of the photon fields.

### 2.4. Radiation Technique

Radiation was delivered on 2–3 consecutive days with 2 Gy doses administered twice daily using 15 MV photons of a linear accelerator (True Beam, Varian Medical Systems, Palo Alto, CA, USA). Patients were placed on a specialized couch 5.45 m away from the gantry with a resulting dose rate of 20 cGy per minute. Four orthogonal lying positions were used for the patient, with an acrylic glass beam spoiler directly in front to ensure a beam build-up effect. In the case of 12 Gy TBI, lungs were blocked in the two lateral positions, limiting the total lung dose to 8 Gy. Additional anterior–posterior opposing fields were used for the mediastinal and axillary regions to enable dose coverage. In vivo dosimetry was performed with semi-conductor probes (PTW, Freiburg, Germany). Eight different measurement points (head, neck, larynx, thorax, mediastinum and abdomen) were used to control for homogeneous delivery, and an additional laryngeal block was placed in case of >10% overdosage.

### 2.5. NTCP Calculation and Replanning

Out of the clinical cohort, 22 patients were randomly assigned to receive NTCP replanning. The lungs and other organs at risk were contoured for all patients by a senior physician in radiation oncology. Subsequently, a dosimetric evaluation for radiological or symptomatic pneumonitis (within the first 6 months after SCT) and lung fibrosis (after 6 months) was carried out using the NTCP model proposed by Lyman–Kutcher–Burman [19].

The Lyman–Kutcher–Burman model is based on a probit function:

$$NTCP_{LKB}=\frac{1}{2\pi }\int_{-\infty }^{t}exp\left(\frac{-u^{2}}{2}\right)du$$

where

$$t=\frac{D_{eff}-D_{50}}{m\cdot D_{50}}$$

and

$$D_{eff}=\sum_{i=1}^{M}\left(\frac{v_{i}}{V_{ref}}EQD_{2,i}^{1/n}\right)²$$

*u* = variable of integration, 𝐷_50_ = dose giving a 50% response probability, *m* = slope of the response curve, *n* = volume dependence, *M* = total number of voxels, *v_i_*/*v_ref_* = relative volume of voxel compared to reference volume and 𝐸𝑄𝐷_2_ = the equivalent dose in voxel when given in 2-gray fractions.

This model employs the parameter “m” for the steepness of the dose–effect curve, “n” to describe volume effects and TD_50_ to account for a 50% risk for the respective side effect. As in [20], the values utilized were 1.02, 0.8 and 0.5 for “n”; 0.26, 0.37, 0.34 for “m” and 21.0, 21.9 and 28.8 for TD_50_ for clinical apparent/symptomatic pneumonitis, all pneumonitis (clinical and radiological) and lung fibrosis, respectively.

## 3. Results

**Figure 1:**
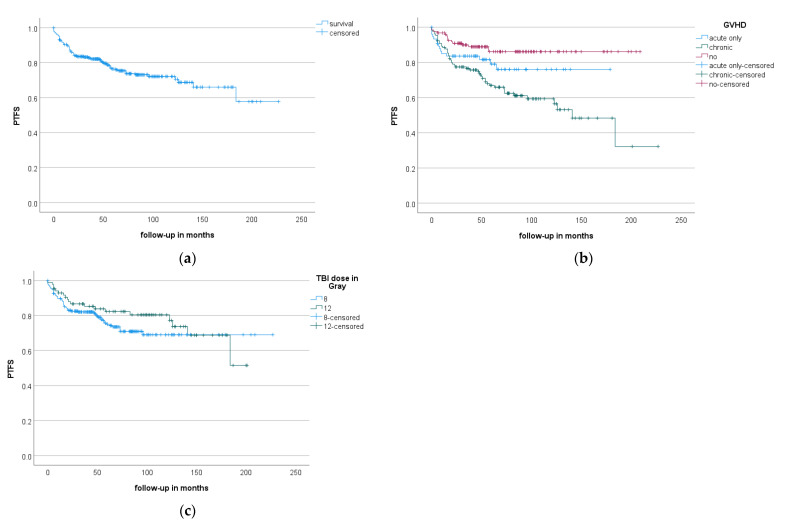
Pulmonary toxicity-free survival (PTFS). (**a**) Kaplan–Meier curve of the whole collective. (**b**) PTFS dependence on occurrence of graft-versus-host-disease (GVHD) for patients suffering from acute (blue), chronic (green) or no (red) GVHD. There was a significant difference in mean PTFS with 141.4 m for acute GVHD, 131.1 m for chronic GVHD and 183.3 m for absence of GVHD (*p* < 0.001), respectively. (**c**) PTFS dependence on the use of total body irradiation (TBI) radiation dose: PTFS percentage for patients treated with 8 (blue) or 12 Gy (green) TBI. There was no significant difference in PTFS (*p* = 0.268).

Overall, 335 patients undergoing TBI were identified (see Table 1 for details), with 219 having acute myeloid leukemia, 98 having acute lymphoid leukemia and 15 having myelodysplastic syndrome. The conditioning chemotherapy regimen consisted of fludarabine in most cases, either alone or in combination with melphalan (243 patients). Patients received TBI with 8 Gray (Gy; 244 patients), 12 Gy (86 patients) or lower doses (5 patients). In total, 330 patients received myeloablative conditioning therapy and 5 patients were treated with reduced intensity conditioning. The study cohort consisted of 192 men (57.3%) and 143 women (42.7%). Median follow-up was 85 months, and the median age at alloSCT was 48 years (50 and 32 years for the 8- and 12-Gy regimens, respectively). During follow-up, 24.8% of all patients displayed some type of pulmonary toxicity, the majority being pneumonia (13.4%), bronchial obstruction (6.0%) or dyspnea, not otherwise specified (2.7%) (Table 2 and Table 3). Diagnosis of pneumonia required the presence of an infectious agent or a pulmonary infection responding to antibiotic treatment, whereas the diagnosis “idiopathic pneumonia syndrome” (IPS) was not found in the clinical files. The majority of toxicities were mild to moderate, being grade 1–2 in 61.4% of patients suffering from pulmonary side effects (Table 2). Overall, the distribution between the different grades was 6.7%, 58.4%, 18.0%, 5.6% and 11.2% for grades 1–5, respectively (Table 3). There were 5 cases of grade 4 pneumonia and 2, 1, 1 and 6 cases of grade 5 acute respiratory distress syndrome (ARDS), bronchial obstruction, pleural effusion and pneumonia, respectively (Table 3). Concerning toxicity grades, the association of different factors with the distribution between grades 1 and 5 was analyzed: neither the type of chemotherapy (*p* = 0.471), RT dose (*p* = 0.690), presence of graft-versus-host-disease (GVHD) (*p* = 0.368) nor age at transplantation (*p* = 0.852) had a significant impact. Tests were repeated including patients without pulmonary toxicity (the remaining patient collective) as grade “0”: GVHD had a significant impact (*p* < 0.001) in contrast to RT dose (*p* = 0.808), type of chemotherapy (*p* = 0.472) and age at transplantation (*p* = 0.675). Pulmonary toxicities appeared 16 months (median) after TBI (Figure 1).

Log-rank analysis on the occurrence of pulmonary toxicity revealed an association of increased risk with the presence of chronic GVHD (Figure 1b: mean PTFS = 141.4 m for acute GVHD, 131.1 m for chronic GVHD, 183.3 m for absence of GVHD; *p* < 0.001).

Furthermore, fludarabine-containing conditioning chemotherapy displayed an elevated risk for pulmonary toxicities in comparison to cyclophosphamide (mean PTFS: 153.4 m vs. 162.8 m, *p* = 0.022). In contrast, neither sex (*p* = 0.313), RT dose (Figure 1c; mean PTFS: 166.9 m vs. 155.9 m for 8 Gy vs. 12 Gy; *p* = 0.268) nor disease entity (*p* = 0.881) were significantly associated with PTFS.

Similar results were seen in the univariate regression analysis, demonstrating an increased risk for pulmonary toxicity in cases with chronic GVHD (relative risk (RR) = 3.34; confidence interval (CI): 1.88–5.95; *p* < 0.001), whereas acute GVHD was not accompanied by a significant risk elevation (*p* = 0.065; Table 4).

A significant decline in risk was observed for patients whose conditioning chemotherapy was based on cyclophosphamide vs. a fludarabine-containing regimen (RR = 0.51; CI: 0.28–0.94; *p* = 0.03), whereas sex (*p* = 0.316), RT dose (*p* = 0.271), age at the time of transplantation (*p* = 0.120) and the chemotherapy comparison of fludarabine vs. fludarabine with melphalan (*p* = 0.288) had no significant impact.

We entered the type of conditioning chemotherapy, presence of acute or chronic GVHD and age at the time of transplantation into the multivariate analysis: only the presence of chronic GVHD (relative risk: 3.31; CI: 1.84–5.95; *p* < 0.001) and the use of cyclophosphamide in the conditioning chemotherapy in comparison to fludarabine (RR: 0.52; CI 0.28–0.96; *p* = 0.036) remained significant.

### NTCP Calculation

Patients in the NTCP planning cohort had a median age of 50.7 years (19.6–70.6 years), a mean lung dose of 7.6 Gy (range: 7.3–8.3 Gy) and revealed a median risk of 20.3% (CI: 19.4–23.3%), 0.6% (CI: 0.5–1.5%) and 20.4% (CI: 19.7–22.8%) for all pneumonitis (clinical and radiological), symptomatic pneumonitis and lung fibrosis, respectively.

## 4. Discussion

The clinical and biophysical data provided in this analysis enable a long-term evaluation of TBI and prove it to be both feasible and safe. The major findings derived from real-world data and the NTCP model are as follows: The estimated NTCP values for pulmonary toxicities fall within the numbers reported in the literature, thereby corroborating the applicability and feasibility of the postulated model. In the clinical cohort, as well as in the NTCP cohort, relevant toxicities occurred in a minority of patients, with only a small subset being high-grade. Nevertheless, some patients displayed grade 4/5 toxicity, which supports the need for specialized treatment units capable of managing life-threatening side effects. Asymptomatic pneumonitis and lung fibrosis are more common but also demand attentive patient care during both initial treatment and follow-up. With a similar rate of overall pneumonitis and lung fibrosis, there might be a conversion from the initial inflammatory to the chronic fibrotic phase in most patients. This idea is supported by the pathophysiological model of radiation-induced lung injury, in which cytokines and growth factors mediate fibroblast proliferation and the aforementioned transition to chronic fibrosis [3,5].

One major advantage of our evaluation is the long follow-up with a median duration of >7 years, which exceeds that of many comparable studies (7.2–32.4 months) [8,9,13,21,22,23]. This is of particular importance as chronic, long-term toxicities such as lung-fibrosis may require months or years to develop [5].

Direct comparison with data from the literature is hampered by the different assessments of pneumonitis, which may be based on clinical data only or takes into account radiological, spirometric and laboratory findings as well [7,9,12,13,14,23,24,25]. NTCP modeling is used to anticipate pulmonary toxicities, although no grading is provided with the calculation presented in Section 2.5. However, the distinction between radiological and symptomatic pneumonitis may be sufficient for clinical application and risk evaluation. Overall, the NTCP model estimated a risk of 20.3% for all pneumonitis types, which is in accordance with the values from the literature (10.3–45% [7,8,9,10,11,12,13,14,15]). Without regular radiological examinations (and search for pneumonitis), the total rate in the clinical cohort could not be evaluated. Symptomatic pneumonitis is supposed to occur in 0.6% of all patients, an incidence comparable to the rate of ARDS in the patient cohort (1.2%). Despite the different pathophysiology, there is an overlapping clinical presentation, and one diagnosis may be mistaken for the other. This is further underlined by the fact that IPS was not found in the clinical files and may have been included as ARDS.

A major challenge for the assessment of lung toxicity is the unspecific symptomatology of dyspnea being caused by anemia, cardiac arrythmia or infections and being further modulated by smoking status, accompanying chemotherapy and radiation schedule [5,6]. Taking this into account, NTCP modeling may be a valuable tool for a priori estimation of side effects, even in the context of large-field techniques such as TBI.

Dose rate, fractionation and total radiation dose are pivotal determinants of radiation pneumonitis [6]. The various dose rates reported in the literature range from 2.5 to 25 cGy/min [7,8,11,12,15,21,22,23,24,26]. Latini et al. suggested a low-dose-rate application to be of cardinal importance for the use of single-fraction TBI to account for the repair of sublethal damage in the lungs [10]. A recent dosimetric evaluation determined a dose rate >15 cGy/min as a significant risk factor for post-transplantation IPS (10% vs. 29% in the first 100 days post-alloSCT) [27]. Other analyses underlined this cut-off value, displaying an odds ratio of 3.36–4.94 for the development of IPS with the use of dose rates ≥15 cGy/min [14,23]. In contrast, a large meta-analysis including 20 studies and 1090 patients failed to identify a significant correlation between dose rate and interstitial pneumonitis [28]. In our department, TBI deliverance is performed with a dose rate of 20 cGy/min, which is a trade-off between possible toxicity and feasibility of application since the delivery still requires a beam-on time of several minutes for each treatment position.

Regarding radiation dose, there was no difference in pneumonitis rate for the 8 and 12 Gy treatment schedules. Assuming an α/β-ratio of 4.0 Gy [6], the resulting biological equivalent doses are 12 Gy (for the 8 Gy regimen) and 18 Gy (for the 12 Gy regimen), the latter being reduced by 33% (6 Gy) by the application of lead lung blocks. Therefore, the virtually identical toxicity rates demonstrate the efficacy of lung blocks and dose reduction. It should be added that data from Oya et al. suggest that a total lung radiation dose of 12 Gy may be administered in fractionated RT without an increase in lung toxicity [13].

Fractionation is a key factor for radiation efficacy in order to account for repopulation and to address cells in different phases of the cell cycle. There is a high variability of fractionation numbers and doses ranging from 10 to 15.6 Gy in 1–12 fractions [8,9,10,11,12,13,14,15,21,22,23,26,29]. A comparison between single and fractionated treatment indicated a decreased rate of interstitial pneumonitis (17.7% vs. 37.5% 5 years after treatment; *p* = 0.02), but an increased rate of disease relapse with a fractionated approach (16% vs. 29% 5 years after treatment; *p* = 0.05) [11]. However, other studies illustrated the feasibility and safety of a hyperfractionated RT schedule [8,9,10,12,21,22,27,30].

Apart from radiation treatment parameters, the incidence of pulmonary toxicity results from a complex interplay of different factors, including age and performance status of the patient, number and type of previous chemotherapies, exposure to infectious agents, disease status before alloSCT, type of GVHD prevention and matching of the transplant via human leukocyte antigens [10,12,22,23,24,28,30]. Corresponding to the latter factors, the presence of a chronic GVHD and chemotherapy type remained significant risk factors for reduced PTFS in the multivariate regression analysis. Additionally, there was a significant association between GVHD and the severity of toxicity (see Results). There have been differing observations on the exact influence of GVHD, as some studies found an increase in pulmonary toxicity risks with overall or (severe) acute [11,15,23,29] forms while others did not identify a significant association [7,12,14].

With the widespread use of intensity-modulated radiation therapy (IMRT), a more selective targeting of lymphoid tissue and bone marrow is made possible. Total marrow and total lymphoid irradiation use IMRT, helical tomotherapy or volumetric arc therapy to avoid surrounding organs at risk (e.g., the lungs), thereby reducing toxicity while enabling dose escalation within the target volume [1,2]. A prospective adaptation of total marrow irradiation achieved a median lung dose of 7 Gy (with 97% of the patients receiving a total dose of 12–19 Gy), with a resulting pneumonitis rate of 0.7% [31]. Another cohort analysis confirmed the reduced toxicity profile of IMRT-based TBI regarding pneumonitis [12]. Despite the presented advantages of modern techniques, benefits have to be weighted against an increased effort in contouring, planning and RT execution with the more conformal approaches [2]. A multi-institutional planning study on total marrow irradiation identified the lungs to be prone to variations in low-dose exposure between the different RT fractions [32].

As a retrospective and monocentric evaluation, the current analysis has some limitations. Previous data on lung diseases and toxin exposure were incomplete, thus not allowing for decisive analysis. With only some patients receiving reduced intensity conditioning before alloSCT, an evaluation of this strategy and the subsequent pulmonary toxicities was not possible. Furthermore, follow-up imaging was not scheduled regularly, which prevented us from evaluating the exact rate of radiologically apparent, but asymptomatic, pneumonitis. It should be pointed out that fludarabine was predominantly used in the 8 Gy cohort in contrast to cyclophosphamide in the 12 Gy group. Consequently, a difference in pulmonary toxicities between the two groups may be masked by the impact of chemotherapy conditioning. Despite the long follow-up, pulmonary toxicities may further rise with longer observation times, although the causative link to TBI appears uncertain. The study may be prone to some bias as only patients with a follow-up of at least 1 year were included to provide adequate data on long-term toxicity. Thus, we did not attempt to calculate survival rates.

## 5. Conclusions

The clinical and biophysical data provided in this analysis demonstrate several key findings. Firstly, the rate of clinically relevant radiation-associated pulmonary side effects is low, both in the risk model and in clinical real-world data, thus corroborating the safety profile of TBI. Secondly, asymptomatic pneumonitis and lung fibrosis are estimated to be more prevalent, which strengthens the need for thorough anamnesis, physical examination and radiological imaging during follow-up. Thirdly, the rate of overall pneumonitis and lung fibrosis is nearly identical, thus supporting the assumption that a majority of patients with (asymptomatic) pneumonitis develop fibrosis during follow-up. Lastly, the NTCP calculation demonstrates the feasibility of the assumed model for clinical application in the setting of TBI as a large-field technique. Further analyses may investigate the impact of non-myeloablative conditioning regimens and modern intensity-modulated TBI approaches on the pulmonary toxicity profile.

## Figures and Tables

**Table 1:** Demographic basic data on clinical collective. ALL: acute lymphoid leukemia; AML: acute myeloid leukemia; Gy: Gray; MDS: myelodysplastic syndrome; PLL: prolymphocytic leukemia; TBI: total body irradiation.

Patient Characteristics	*n* (% or Range)
Number of patients	335
Median age at transplantation	48 (18–74)
Sex	
Male	192 (57.3)
Female	143 (42.7)
Diseases	
AML	219 (65.4)
ALL	98 (29.3)
T-cell ALL	22 (22.4 of ALL)
B-cell ALL	76 (77.6 of ALL)
MDS	15 (4.5)
Biphenotypic leukemia	2 (0.6)
T-PLL	1 (0.3)
Chemotherapy regimen	
Fludarabine	147 (43.9)
Melphalan-fludarabine	96 (28.7)
Cyclophosphamide	74 (22.1)
Etoposide	11 (3.3)
Other	7 (2.1)
Graft-versus-host-disease	
No	124 (37.0)
Acute only	75 (22.4)
Chronic	131 (39.1)
No information	5 (1.5)
TBI dose	
8 Gy	244 (72.8)
12 Gy	86 (25.7)
<8 Gy	5 (1.5)

**Table 2:** Absolute numbers and percentages of patients regarding pulmonary toxicities and grade of toxicities. Multiple toxicities were possible for each patient. ARDS: acute respiratory distress syndrome.

Pulmonary Toxicity	*n* (% or Range)
Type of toxicity	
Overall	83 (24.8)
Pneumonia	45 (13.4)
Bronchial obstruction	20 (6.0)
Dyspnea	9 (2.7)
Pleural effusion	7 (2.1)
ARDS	4 (1.2)
Other	4 (1.2)
Maximum grade of toxicity	
Grade 1–2	51 (61.4)
Grade 3–5	32 (38.6)

**Table 3:** Overview of pulmonary toxicities and their respective grades. Percentage numbers in parentheses indicate the fraction of the respective grade for a given toxicity. ARDS: acute respiratory distress syndrome.

Type of Toxicity	Grade 1	Grade 2	Grade 3	Grade 4	Grade 5	Total
ARDS	0 (0.0%)	0 (0.0%)	2 (50.0%)	0 (0.0%)	2 (50.0%)	4 (100.0%)
Bronchial obstruction	1 (5.0%)	15 (75.0%)	3 (15.0%)	0 (0.0%)	1 (5.0%)	20 (100.0%)
Dyspnea	5 (55.6%)	4 (44.4%)	0 (0.0%)	0 (0.0%)	0 (0.0%)	9 (100.0%)
Lung edema	0 (0.0%)	1 (100.0%)	0 (0.0%)	0 (0.0%)	0 (0.0%)	1 (100.0%)
Pleural effusion	0 (0.0%)	2 (28.6%)	4 (57.1%)	0 (0.0%)	1 (14.3%)	7 (100.0%)
Pneumonia	0 (0.0%)	27 (60.0%)	7 (15.6%)	5 (11.1%)	6 (13.3%)	45 (100.0%)
Pneumothorax	0 (0.0%)	1 (100.0%)	0 (0.0%)	0 (0.0%)	0 (0.0%)	1 (100.0%)
Pulmonary hypertension	0 (0.0%)	1 (100.0%)	0 (0.0%)	0 (0.0%)	0 (0.0%)	1 (100.0%)
Vital capacity decrease	0 (0.0%)	1 (100.0%)	0 (0.0%)	0 (0.0%)	0 (0.0%)	1 (100.0%)
Total	6 (6.7%)	52 (58.4%)	16 (18.0%)	5 (5.6%)	10 (11.2%)	89 (100%)

**Table 4:** Overview on relative risk according to univariate and multivariate analyses. Values of relative risk were rounded to two decimal places. ALL: acute lymphoid leukemia; AML: acute myeloid leukemia; cyc: cyclophosphamide; flu: fludarabine; GVHD: graft-versus-host-disease; Gy: Gray; mel: melphalan; MDS: myelodysplastic syndrome; RR: relative risk; RT: radiotherapy; SCT: stem cell transplantation.

Variable	Comparison	Univariate Analysis			Multivariate Analysis (Step 1)			Multivariate Analysis (Step 2)		
		RR	Range	*p*	RR	Range	*p*	RR	Range	*p*
GVHD	acute vs. none	1.97	0.96–4.04	0.065	1.92	0.93–3.95	0.076	1.90	0.92–3.91	0.081
	chronic vs. none	3.34	1.88–5.95	≤0.001	3.33	1.85–5.99	≤0.001	3.31	1.84–5.95	≤0.001
Conditioning chemotherapy	cyc vs. flu	0.51	0.28–0.94	0.030	0.59	0.29–1.16	0.126	0.52	0.28–0.96	0.036
	flu and mel vs. flu	0.75	0.45–1.27	0.288						
Disease	ALL vs. AML	0.97	0.60–1.57	0.895						
	MDS vs. AML	0.75	0.23–2.38	0.621						
Sex	male vs. female	1.26	0.81–1.96	0.316						
RT dose	8 Gy vs. 12 Gy	1.34	0.80–2.26	0.271						
Age at SCT		1.01	1.00–1.03	0.120	1.01	0.99–1.03	0.433			

## Data Availability

The data presented in this study are available in the article.
